# The loss of a supergene in obligately polygynous *Formica* wood ant species

**DOI:** 10.1093/molbev/msaf320

**Published:** 2025-12-16

**Authors:** Hanna Sigeman, Ina Satokangas, Matthieu de Lamarre, Patrick Krapf, Pierre Nouhaud, Riddhi Deshmukh, Heikki Helanterä, Michel Chapuisat, Jonna Kulmuni, Lumi Viljakainen

**Affiliations:** Ecology and Genetics Research Unit, University of Oulu, Oulu, Finland; Department of Medical Biochemistry and Microbiology, Uppsala University, Uppsala, Sweden; Organismal and Evolutionary Biology Research Programme, University of Helsinki, Helsinki, Finland; Department of Ecology and Evolution, University of Lausanne, Lausanne, Switzerland; Organismal and Evolutionary Biology Research Programme, University of Helsinki, Helsinki, Finland; Institute for Biodiversity and Ecosystem Dynamics, University of Amsterdam, Amsterdam, Netherlands; Organismal and Evolutionary Biology Research Programme, University of Helsinki, Helsinki, Finland; UMR CBGP, INRAE, CIRAD, IRD, Institut Agro, Université Montpellier, Montpellier, France; Department of Ecology and Evolution, University of Lausanne, Lausanne, Switzerland; Department of Medical Biochemistry and Microbiology, Uppsala University, Uppsala, Sweden; Department of Ecology and Evolution, University of Lausanne, Lausanne, Switzerland; Organismal and Evolutionary Biology Research Programme, University of Helsinki, Helsinki, Finland; Institute for Biodiversity and Ecosystem Dynamics, University of Amsterdam, Amsterdam, Netherlands; Ecology and Genetics Research Unit, University of Oulu, Oulu, Finland

**Keywords:** supergene, *Formica*, polygyny, ants

## Abstract

Some of the most striking examples of phenotypic variation within species are controlled by supergenes. However, most research on supergenes has focused on their emergence and long-term maintenance, leaving the later stages of their life cycle largely unexplored. Specifically, what happens to a derived supergene haplotype when the trait it controls reaches fixation? Here we answer this question using the ancient supergene system of *Formica* ants, where (monogynous) single-queen colonies typically carry only the ancestral haplotype M while the derived haplotype P is exclusive to (polygynous) colonies with multiple queens. Through comparative population genomics of 264 individuals from all seven European wood ant species, we found that the P haplotype was present in only 1/3 obligately polygynous species (*Formica polyctena*). In the two others (*Formica aquilonia* and *Formica paralugubris*), the P haplotype was completely missing except for duplicated P-specific paralogs of two genes, *Zasp52* and *TTLL2*, with *Zasp52* being directly involved in wing muscle development. We hypothesize that these genes play a direct role in polygyny and contribute to differences in body size and/or dispersal behavior between monogynous and polygynous queens. A complete lack of P/P genotypes among the 261 workers suggests strong selection against such genotypes. While our analyses did not reveal evidence of increased mutation load on the P, it is possible that this skew in genotype distributions is driven by a few loci with strong fitness effects. We propose that selection to escape P-associated fitness costs underlies the loss of this haplotype in obligately polygynous wood ants.

## Introduction

Supergenes underlie some of the most dramatic within-species polymorphisms in nature, such as color morphs in mimetic butterflies (Kimura 1956; Clarke and Sheppard 1960; Joron et al. 2006), male plumage morphs in birds (Küpper et al. 2016; Lamichhaney et al. 2016), and colony social organization in ants (Wang et al. 2013; Purcell et al. 2014; Brelsford et al. 2020). They often form through structural rearrangements which block recombination with the ancestral haplotype and can be selectively beneficial and rise in frequency if they capture multiple co-adapted alleles (Kimura 1956; Turner 1967; Thompson and Jiggins 2014; Charlesworth 2016). Novel, or derived, supergene haplotypes are likely to have either higher or lower fitness than the ancestral haplotype, leading to their fixation or elimination (Schwander et al. 2014). However, supergenes can also exist as stable polymorphisms maintained by balancing selection for millions of years and across numerous speciation events (Wang et al. 2013; Purcell et al. 2014, 2021; Brelsford et al. 2020). Empirical work on supergenes is predominantly aimed at understanding the selection regimes and molecular dynamics underlying their emergence and maintenance in species with phenotypic variation, leaving the later stages of their evolutionary trajectory largely unexplored. Specifically, what happens to a supergene system in species where the phenotypic polymorphism it controls has disappeared?

In species where the derived trait has become fixed, one might intuitively also expect (i) fixation of the derived haplotype, as seems to have happened in two species of mimetic swallowtail butterflies (Zhang et al. 2017). However, this is not possible in all supergene systems since derived haplotypes often become degenerated due to inefficient selection against deleterious mutations, leading to strong negative fitness effects or even homozygous lethality (Schwander et al. 2014; Llaurens et al. 2017; Jay et al. 2021). Alternative outcomes are then to either (ii) maintain the supergene polymorphism despite lacking the phenotypic variation, as shown possible from population genetic models (Tafreshi et al. 2022), or to (iii) retain only a subset of genes from the derived haplotype needed to uphold the derived trait, while otherwise reverting to fixation of the ancestral haplotype. This last outcome would allow purging of deleterious mutations accumulated on the derived haplotype. Here, we test between these molecular outcomes using the ancient supergene system of *Formica* ants (∼20 to 40 my; Brelsford et al. 2020; Purcell et al. 2021).

Socially polymorphic ants, including many *Formica* species, display intra-species variation in reproductive queen number, with some colonies being headed by a single queen (monogyny) while others contain multiple, often non-related, queens (polygyny) (Sundström 1993; Goropashnaya et al. 2001; Chapuisat et al. 2004; DeHeer and Herbers 2004; Seppä et al. 2004; Rosset and Chapuisat 2007). In socially polymorphic ants, queen number is associated with a suite of behavioral, phenotypic, and life-history traits, collectively referred to as the “polygyny syndrome” (sensu Keller 1993). These include differences in colony size, queen and worker body size, and dispersal behavior (Keller 1991; Rosset and Chapuisat 2007). The polygyny syndrome in *Formica* is genetically controlled by a ∼10 Mb supergene on chromosome 3, which formed through multiple inversion events in a shared ancestor (Purcell et al. 2014; Brelsford et al. 2020). In monogynous *Formica* colonies, individuals generally carry only the ancestral haplotype M (diploid workers and queens are M/M; haploid males are M), while the derived P haplotype is present within polygynous colonies though not necessarily in every individual (Purcell et al. 2014; Brelsford et al. 2020; Brelsford et al. 2020; Lagunas-Robles et al. 2021; McGuire et al. 2022; Pierce et al. 2022). While this association between colony social organization and supergene haplotypes is generally strong across *Formica* species, recent studies have found some colonies of *Formica aserva* and *Formica cinerea* that deviate from these patterns (Scarparo et al. 2023, 2024). The P haplotype carries genes adapted to polygyny, but is also associated with recessive fitness costs. This is evidenced by lower survival rates of P/P females in *Formica selysi* compared to other genotypes (Blacher et al. 2023) and further suggested by a complete or near lack of P/P workers in several *Formica* species (Lagunas-Robles et al. 2021; Purcell et al. 2021; Pierce et al. 2022; Scarparo et al. 2024).

Here we are investigating the evolutionary fate of this supergene across all seven European species of the *Formica rufa* (“wood ants” or *Formica s.str* group) complex. The group includes four socially polymorphic species (*Formica lugubris*, *Formica pratensis*, *F. rufa*, and *Formica truncorum*) and three which are commonly described as obligately polygynous (*Formica aquilonia*, *Formica paralugubris*, and *Formica polyctena*). While it is possible that polygyny is not completely fixed across the entire range of these three species (see e.g. Lagunas-Robles et al. (2025)), especially given the occasional hybridization between socially polymorphic and obligately polygynous wood ants (Monaghan 2022; Satokangas et al. 2023; Järvinen et al. 2025), they are nonetheless almost ubiquitously reported as such. *F. aquilonia* and *F. paralugubris* are highly polygynous species with queen numbers frequently reaching several hundred per nest (Pamilo 1982; Rosengren and Pamilo 1983; Pamilo et al. 1992, 2005; Chapuisat et al. 1997; Chapuisat and Keller 1999; Mäki-Petäys et al. 2005; Sundström et al. 2005; Holzer et al. 2009; Vanhala et al. 2014; Seifert 2018; Masoni et al. 2022). *Formica polyctena* have been described as invariably polygynous (Sundström et al. 2005) or almost completely polygynous (Seifert et al. 2010), but with a lower queen number than in *F. aquilonia* (Pamilo 1982; Gyllenstrand et al. 2004). Using comparative population genomic analyses of 264 ants, we show that the molecular outcome (see above; 1 to 3) of the supergene system varies between the obligately polygynous species. We also show that species in which a derived supergene-associated trait has become fixed are powerful study systems for revealing genes involved in these traits.

## Results and discussion

### Obligately polygynous *Formica* species lack the polygyny-associated haplotype P

**Figure 1:**
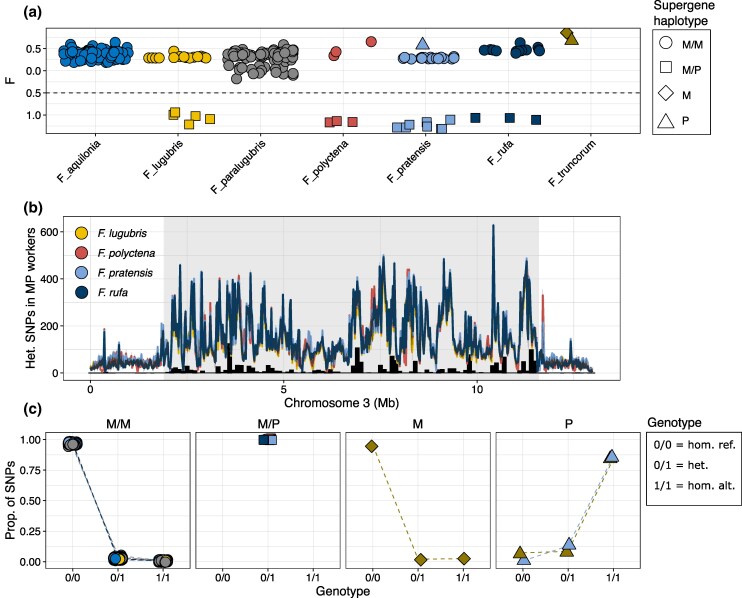
a) Inbreeding coefficients (F_IS_ scores) for all samples (*n* = 261 diploid workers, *n* = 3 haploid males). The shape of the data points shows the supergene haplotype, as revealed by the results in a), b), and c). The 19 workers with inbreeding coefficients <−0.5 (below the dashed line) were classified as M/P heterozygotes. b) The lines show mean number of heterozygous SNPs (20 kb windows) per species, for the 19 M/P workers (*n* = 5 *F. lugubris*, *n* = 8 *F. pratensis*, *n* = 3 *F. polyctena*, and *n* = 3 *F. rufa*). The supergene region (1.9 to 11.6 Mb) is highlighted with a gray background color. The black histogram shows the number of sites that were heterozygous in all 19 workers (“trans-species haplotype-specific SNPs”; *n* = 21,643). c) Relative proportions of genotype counts (0/0 = reference homozygous, 0/1 = heterozygous, 1/1 = alternative homozygous) across the 21,643 trans-species haplotype-specific SNPs for each individual (i.e. the total value for each individual amount to 1.00). A dashed line connects the data points from the same individual. The shape and color of the data points are consistent with a).

We determined supergene genotype distributions across the seven *Formica* species using whole-genome sequencing data of 264 individuals (Table S1) with an average sequencing depth of 21.9× (Table S2). Diploid individuals (workers and queens) with M/P supergene genotypes are expected to display high heterozygosity levels across the supergene region on chromosome 3. Of the 261 workers in our dataset, 19 were deemed as highly heterozygous based on inbreeding coefficients (F_IS_ scores <−0.5; Fig. 1a; Table S3). The heterozygous SNPs in these workers were concentrated within a 10.7 Mb central region of chromosome 3 positioned between 1.9 and 11.6 Mb (Fig. 1b; Figure S1). This region is highly syntenic to the supergene region in other *Formica* species (Brelsford et al. 2020; Lagunas-Robles et al. 2021; Purcell et al. 2021), confirming their status as M/P heterozygotes (*n* = 5 *F. lugubris*, *n* = 8 *F. pratensis*, *n* = 3 *F. polyctena*, and *n* = 3 *F. rufa*).

The genotypes of the remaining workers (*n* = 242; F_IS_ scores >−0.5; Fig. 1a; Table S3) were determined based on their allele distribution at the 21,643 sites on chromosome 3 which were heterozygous across all 19 workers with M/P genotypes (referred to as “trans-species haplotype-specific SNPs”; Fig. 1b and c) and by the inclusion of three (haploid) males from a previous study with known supergene status (Table S1; Brelsford et al. 2020). All non-M/P workers were almost exclusively homozygous for the reference allele (Fig. 1c; Table S4), similarly to the *F. truncorum* male with a known M genotype (Fig. 1c; Table S4), and therefore classified as M/M homozygotes. These results also show that the reference genome, which was constructed from a single male from a *F. aquilonia*  *×*  *polyctena* hybrid population in southern Finland (Nouhaud et al. 2022), contains the M haplotype. This conclusion is further supported by the *F. pratensis* and *F. truncorum* males with known P genotypes, which both had the alternative allele across almost all sites (Fig. 1c; Figures S1 and S2; Table S4).

**Figure 2:**
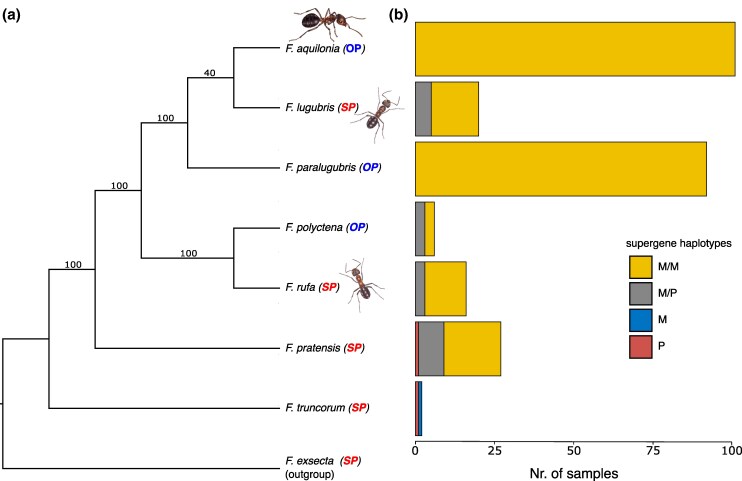
a) Phylogeny of the seven studied species, including an outgroup (*F. exsecta*, also carrying the supergene), based on SNPs from chromosome 3 outside the supergene region (see Methods). Bootstrap values are displayed as branch labels. OP, obligately polygynous; SP, socially polymorphic. b) The distribution of supergene genotypes for each species (Table S5). Ant drawings by Lizzie Harper.

Counter-intuitively, the polygyny-associated P haplotype was completely lacking from two of the three obligately polygynous species (*F. aquilonia* and *F. paralugubris*; Fig. 2; Table S5). This is a surprising finding as the species share multiple polygyny syndrome characteristics assumed to be linked to the P haplotype, such as high queen number per colony, large colony sizes, and colony formation mainly through budding (Seifert 2018). While we lack direct evidence on colony social structure for the workers in this study, we can safely assume that the vast majority, or all, of these M/M workers (Fig. 2) originate from polygynous colonies. *F. aquilonia* is a supercolonial species known to be highly polygynous across widely sampled European populations (Pamilo 1982; Rosengren and Pamilo 1983; Pamilo et al. 1992, 2005; Mäki-Petäys et al. 2005; Sundström et al. 2005; Seifert 2018), and the workers in this study were collected from numerous colonies over a wide geographical range (*n* = 101; Austria, Finland, Italy, Scotland, and Switzerland; Table S1). Additionally, for the 20 *F. aquilonia* samples from the Nenännokka population (Table S1), we have visual confirmation of multiple queens for each colony. The *F. paralugubris* workers were collected from a single population in Switzerland (*n* = 92; Table S1), where their highly polygynous social organization has been well characterized (Pamilo et al. 1992; Chapuisat et al. 1997; Chapuisat and Keller 1999; Holzer et al. 2009). While we cannot rule out the presence of the P haplotype across the entire species range of *F. aquilonia* and *F. paralugubris*, our results indicate that its absence is widespread and likely represents a species-level, or near-species-level, pattern. The absence of P in these species shows that fixation of the polygyny syndrome did not occur through fixation of the polygyny-associated haplotype (thus ruling out “molecular outcome 1”; see Introduction). This also suggests that the ancient and otherwise stable *Formica* supergene polymorphism is absent in these obligately polygynous species (i.e. effectively ruling out “molecular outcome 2”).

In contrast, the P haplotype was found within individuals of the obligately polygynous species *F. polyctena* (*n* = 3/6; Fig. 2b; Table S5; i.e. “molecular outcome 2”) and within all four socially polymorphic species (*n* = 5/20 *F. lugubris*, *n* = 8/27 *F. pratensis*, *n* = 3/16 *F. rufa*, and *n* = 1/2 *F. truncorum*; Fig. 2b; Table S5). It is likely that the individuals from socially polymorphic species carrying P haplotypes originate from polygynous colonies, since the P haplotype is generally associated with polygyny (but see Brelsford et al. (2020) and Scarparo et al. (2023, 2024) for exceptions to this rule). Note that the polygynous colony social structure is confirmed for the two males with P haplotypes (Fig. 2b; Table S5; Brelsford et al. 2020). Based on the supergene haplotype distributions in other *Formica* species, the colony social structure associated with the M/M genotypes cannot be inferred with certainty (Fig. 2), as M/M genotypes have been found in monogynous as well as polygynous colonies of some *Formica* species (McGuire et al. 2022; Scarparo et al. 2023, 2024).

A parallel study similarly reported a complete absence of the P haplotype among workers from 29 *F. paralugubris* nests (Lagunas-Robles et al. 2025). In contrast to our findings, they detected the P haplotype in one of four *F. aquilonia* nests. As three of these four nests were classified as monogynous (Lagunas-Robles et al. 2025), a social structure highly atypical for this species, we suggest that these samples may have originated from a hybrid population (see Supplementary Note for more details). Further investigation will be needed to clarify their taxonomic status and whether the P haplotype is present at low frequencies within non-admixed populations of *F. aquilonia*.

### Two candidate genes for the maintenance of polygyny

So how is the polygyny syndrome maintained in *F. aquilonia* and *F. paralugubris* despite lacking the P haplotype? One explanation would be if a subset of P-specific gene copies necessary for upholding polygyny remains on chromosome 3 in these species (“molecular outcome 3”), which could be achieved through double crossing over or gene conversion between the supergene haplotypes. This hypothesis is plausible in the *Formica* system as rare recombination events are known to have homogenized varying parts of the M and P haplotypes within different socially polymorphic species (Brelsford et al. 2020). Previous studies have suggested that genes containing trans-species P-specific SNPs (i.e. which have not experienced recombination between haplotypes) are especially likely to play a functional role in maintaining the polygyny syndrome in *Formica* ants (Brelsford et al. 2020; Purcell et al. 2021). We therefore searched for genes with elevated numbers of such P-specific alleles (defined as alternative homozygous or heterozygous SNPs) in obligately polygynous M/M workers compared to socially polymorphic M/M workers. This is because we do not expect the M haplotype in socially polymorphic species to contain genes coding for polygynous traits.

**Figure 3:**
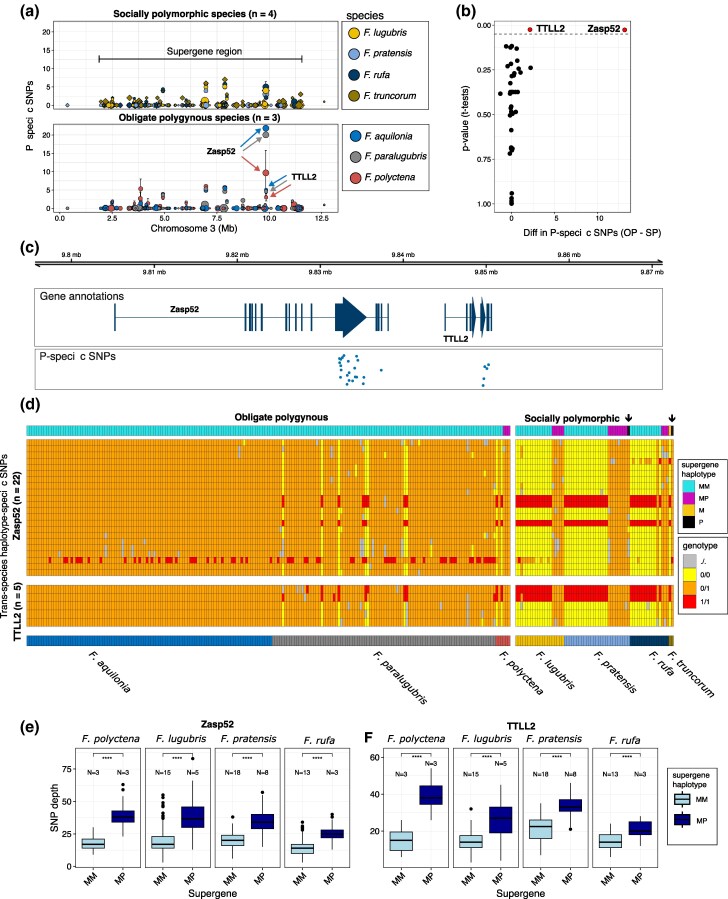
a) Mean number of P-specific SNPs per gene and species (error bars represent ±SE), plotted separately for socially polymorphic (top panel) and obligately polygynous (bottom panel) species. b) *x* axis: Differences in mean number of P-specific SNPs for socially polymorphic (SP) and obligately polygynous (OP) species. *y* axis: *P*-values from *t*-tests between the number of P-specific SNPs in socially polymorphic and obligately polygynous species. The dashed line marks the 0.05 significance level, and genes with significant differences are labeled and colored in red. c) Gene annotations and genome positions of the two genes, Zasp52 and TTLL2, with significantly more P-specific SNPs in the obligately polygynous species. Data points for the P-specific SNPs are shown below, jittered along the *y* axis for increased visibility. d) Heatmap showing from the top: (i) supergene haplotype, (ii) genotypes at the trans-species haplotype-specific SNPs of the genes Zasp52 and TTLL2, and (iii) species. The black arrows highlight the two P males (*n* = 1 *F. pratensis*, *n* = 1 *F. truncorum*), which both appear completely heterozygous despite being haploid, suggesting P-specific duplications of these genes. e and f) Boxplots contrasting depth values for SNPs shown in e), between M/M and M/P workers from all species with both supergene genotypes. The *N* value represents the number of samples for each boxplot. Significance levels from Wilcoxon tests (all *P* < 0.001) are shown for the two genes, e) Zasp52 and f) TTLL2.

We found P-specific copies of only two genes, *Zasp52* (“Z band alternatively spliced PDZ-motif protein 52”) and *TTLL2* (“Tubulin Tyrosine Ligase Like 2”), in M/M workers of obligately polygynous species but not in M/M workers of socially polymorphic species (Fig. 3). Both genes, which are adjacently positioned (9.80−9.85 Mb; Fig. 3c), had significantly more P-specific SNPs in the obligately polygynous species (*Zasp52*: *n* = 22 SNPs, two-tailed *t*-test: *P* = 0.026; *TTLL2*: *n* = 5 SNPs; two-tailed *t*-test: *P* = 0.025; Fig. 3a and b; Tables S6 and S7). After Bonferroni correction for the number of tests (*n* = 283), all *P*-values obtained the value of 1. The SNPs within these genes were almost completely heterozygous in all M/M workers of *F. aquilonia* (*n* = 101/101), most M/M workers of *F. paralugubris* (*n* = 85/92) and at lower frequencies in M/M workers of *F. polyctena* (*n* = 1/3), similarly to all 19 M/P workers of the socially polymorphic species (Fig. 3d). In contrast, the M/M workers of the socially polymorphic species were mainly homozygous for the reference (i.e. ancestral) allele, except for one *F. rufa* M/M worker who was also highly heterozygous (Fig. 3d). Note also that the M/M workers of the socially polymorphic species were mainly alternative homozygous at 5 of the 27 trans-species haplotype-specific SNPs, suggesting that the M haplotype in the *F. aquilonia*  *×*  *polyctena* reference genome contain some P-specific alleles (Fig. 3d).

We propose that the P-specific copies of *Zasp52* and *TTLL2* on the M haplotype may be responsible for upholding the polygyny syndrome in *F. aquilonia* and *F. paralugubris*. *Zasp52* is a well-studied central gene for muscle tissue development in insects, specifically involved in the formation and maintenance of Z-discs which act as boundaries between muscle sarcomeres (Chechenova et al. 2013; Katzemich et al. 2013; Liao et al. 2016). It is reasonable to imagine that such a gene could be involved in the polygyny syndrome, as studies of socially polymorphic species have shown that queens from polygynous colonies are generally smaller and disperse over shorter distances than those from monogynous colonies. Interestingly, flight muscles of polygynous *F. truncorum* queens have fewer mitochondria and sarcomeres and may deteriorate faster (shown by heavily distorted Z-discs in dealate queens) than those of monogynous queens (Johnson et al. 2005). Note, however, that in other (non-wood ant) *Formica* species, polygyny is significantly associated with higher muscle mass in males but not in queens (*F. exsecta*), or not associated with muscle mass in either sex (*Formica pressilabris*; Hakala 2020). *TTLL2* has not been studied in insects but is in humans almost exclusively expressed in sperm (Fagerberg et al. 2014).

**Figure 4:**
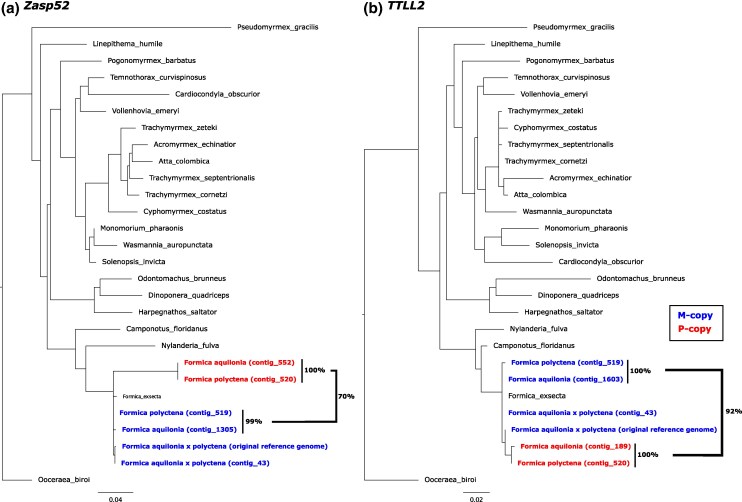
Amino acid gene trees of a) Zasp52 and b) TTLL2. Gene sequences are from the (1) *F. aquilonia* × *polyctena* reference genome, de novo assemblies of PacBio data from males of (2) *F. aquilonia*, (3) *F. polyctena*, and the (4) *F. aquilonia* × *polyctena* reference genome individual, as well as (5) 22 outgroup ant species. The original (blue) and duplicated (P-specific; red) gene copies from the *F. rufa* complex species are highlighted, as well as the nucleotide sequence similarities (%) between some of the gene copies.

Combined evidence suggests that a duplication event created paralogs of *Zasp52* and *TTLL2* on the P haplotype in a shared ancestor of all wood ants and that these P-specific gene copies later translocated to the M haplotype in the obligately polygynous species. Firstly, the two male samples with P haplotypes (*F. pratensis* and *F. truncorum*) are completely heterozygous for all trans-species haplotype-specific SNPs within these two genes (Fig. 3d). This points toward a P-specific duplication of these genes, as (haploid) males cannot be truly heterozygous (see e.g. Purcell et al. (2021)). This is further supported by heightened sequencing depth values within this region in individuals with high heterozygosity (Fig. 3e and f; Figs. S3 and S4). Secondly, the high number of trans-species haplotype-specific SNPs shared by the M/P workers as well as the P males suggests that the duplication occurred in a shared ancestor of all wood ants (Fig. 3d). And thirdly, de novo genome assemblies based on *F. aquilonia* and *F. polyctena* males with M haplotypes (see Methods; Tables S8 and S9) revealed two paralogs each of *Zasp52* and *TTLL2*, on separate contigs (Table S10), indicating translocation of the P-specific copies to the M haplotype background in obligately polygynous wood ants (Fig. 4). In both genome assemblies, one gene copy was highly similar to the gene copy in the *F. aquilonia*  *×*  *polyctena* reference genome annotation and to the closest non-wood ant relative *F. exsecta*. These were classified as the original gene copies (Fig. 4; tip labels in blue). The other gene copy in the *F. aquilonia* and *F. polyctena* genomes, which were highly similar to each other, shared nucleotide sequence similarity to their paralogs of 70% (*Zasp52*) and 92% (*TTLL2*). These were classified as the duplicated, P-specific, paralogous gene copies (Fig. 4; in red). The functionality of both gene copies was confirmed by gene expression using RNA data from 400 pooled *F. aquilonia* workers (see Methods; Fig. S5).

Since the species carrying both gene copies of *Zasp52* and *TTLL2* on the M haplotype (the majority of workers in *F. aquilonia*, *F. paralugubris*, and *F. polyctena*, as well as one *F. rufa* worker) are not monophyletic (Fig. 2), we find the most parsimonious interpretation of these patterns to be that a recombination event between the M and P haplotype in one of the three obligately polygynous species (most likely *F. aquilonia* or *F. paralugubris*, see below) created a “minimal P” haplotype, specifically a M haplotype containing the P-specific copies of *Zasp52* and *TLLL2*, followed by adaptive introgression of this new haplotype to the other species (see e.g. Stolle et al. (2022)). Alternatively, the translocation could have originated in a shared ancestor of the closely related species *F. aquilonia* and *F. paralugubris* (Fig. 2), in which it is found in high frequencies (*F. aquilonia*: *n* = 64/64; *F. paralugubris*: *n* = 15/19) and more recently introgressed to *F. polyctena* in which the translocation occurs at lower frequencies (*n* = 1/3 M/M workers). A third and less likely scenario is if the translocation occurred more basally in the group, e.g. after the split between *F. pratensis* and the other wood ant species, followed by repeated losses across the socially polymorphic species. The P-specific duplication was not found among six individuals belonging to five outgroup *Formica* species carrying P haplotypes (*n* = 1 P/P *F. exsecta*, *n* = 1 P/P *F. cinerea*, *n* = 2 P/P *Formica lemani*, *n* = 1 M/P *Formica obscuripes*, *n* = 1 P *F. selysi*; Table S1; Purcell et al. 2021), as there were no heterozygous SNPs within these genes.

### The Knockout gene does not maintain polygyny in *F. aquilonia* and *F. paralugubris*

Previous research on the *Formica* supergene system has revealed other genes hypothesized to play a role in the polygyny syndrome. The main candidate is the gene *Knockout* (Table S6), where multiple trans-species haplotype-specific SNPs are conserved throughout large parts of the *Formica* phylogeny (Purcell et al. 2021). In wood ants, 19 SNPs within the coding region of *Knockout* were similarly trans-species haplotype-specific, but none were P-specific in the two obligately polygynous species lacking P haplotypes (*F. aquilonia* and *F. paralugubris*; Fig. S6). It is therefore unlikely that *Knockout* is crucial for upholding the polygyny syndrome in these species. We also identified single-copy orthologs to four of the six additional candidate genes described in Purcell et al. (2021), all harboring large numbers of trans-species haplotype-specific SNPs between a wide range of *Formica* species (*AmGR10*, *FMFFaR*, *Single-minded*, and *ZPF148*; Table S6). Among the three genes that displayed trans-species haplotype-specific SNPs also within the wood ants (*AmGR10*: *n* = 12; *Single-minded*: *n* = 11; *ZPF148*: *n* = 4), no SNPs were P-specific in *F. aquilonia* or *F. paralugubris* (Figure S6). Note that *Zasp52* and *TTLL2* have not been proposed as candidate genes underlying the polygyny syndrome in other *Formica* species, suggesting that the genetic architecture of polygyny may vary across the group.

### P/P lethality and mutation load as drivers behind the loss of the P haplotype

Three alternative molecular outcomes for a derived supergene haplotype in species where the trait controlled by this haplotype has reached fixation were previously outlined: (i) fixation of the derived P haplotype, (ii) maintenance of the supergene polymorphism, or (iii) retention only of a subset of P-specific genes needed to uphold the derived trait. We found that these molecular outcomes vary between the obligately polygynous species in this study. In *F. polyctena*, the supergene polymorphism remains (molecular outcome 2), while the derived supergene haplotype P has become limited to only a few genes in *F. aquilonia* and *F. paralugubris* (molecular outcome 3). Similarly to previous studies on several other *Formica* ants (Lagunas-Robles et al. 2021; Pierce et al. 2022; Scarparo et al. 2024), we did not find any P/P homozygotes among the 261 workers in our dataset. This suggests that P/P homozygotes are either unviable or subject to strong negative selection in some lineages (Purcell et al. 2021; Blacher et al. 2023). Based on these observations, it is likely that selection against P/P genotypes has prevented fixation of the P haplotype within obligately polygynous *Formica* wood ants (molecular outcome 1). Strong selection against P/P genotypes would also affect the fitness of M/P queens mating with a P male. Such effects were demonstrated in *F. selysi*, one of the few *Formica* species where P/P individuals have been found, where M/P and P/P queens laid smaller broods and had a smaller proportion of offspring reaching adulthood compared to M/M queens (Blacher et al. 2023). Interestingly, the study further showed that fewer M/P and P/P queens were fertile compared to M/M queens and that M/P and P/P workers had a lower survival probability compared to M/M workers (Blacher et al. 2023). This suggests a mutation load for the P haplotype also when occurring in heterozygous form and may contribute to why a recombinant supergene haplotype in wood ants (molecular outcome 3) containing only a subset of genes from the P haplotype could have reached high frequencies (in *F. paralugubris*) and even fixation (in *F. aquilonia*).

**Figure 5:**
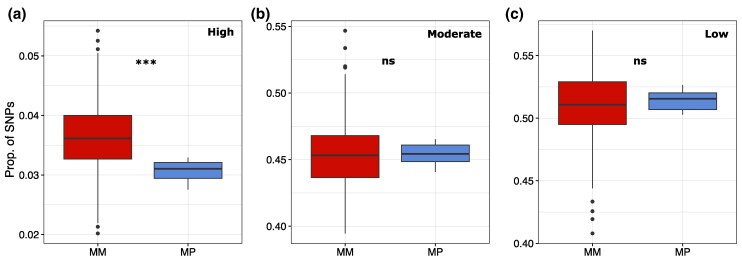
Relative proportions of SNPs with predicted effects on gene functions: a) high (e.g. frame-shift variants, whole-exon deletions, stop codons), b) moderate (e.g. non-synonymous mutation, in-frame deletion, in-frame insertion), and c) low (e.g. synonymous substitution). Only SNPs within the supergene region on chromosome 3 (1.9 to 11.6 Mb) are included. The line within each boxplot shows median values across M/M workers (*n* = 242) and M/P workers (*n* = 19). The number of SNPs for each category and sample is also provided in Table S11.

To estimate whether the wood ant P haplotype carries a mutation load, we predicted the functional effect of all intragenic SNPs within the supergene region of chromosome 3 (categories “high”, “moderate”, and “low”; see Fig. 5 legend for descriptions of each category). The proportion of high- and moderate-impact mutations was not higher in the M/P workers than in the M/M workers (Fig. 5c and d), as would have been expected from a P haplotype carrying a high mutation load. Contrary to expectations, the M/M workers had a significantly higher proportion of high-impact mutations compared to the M/P workers (Fig. 5a; Wilcoxon: *W* = 3861, *P* = <0.001), while there was no difference between the proportions of moderate- (Fig. 5b; Wilcoxon: *W* = 2210, *P* = 0.78) or low-impact mutations (Fig. 5c; Wilcoxon: *W* = 2022, *P* = 0.3828). It is possible that recombination or gene conversion events between the M and P haplotypes, as observed within *Formica* ants (Brelsford et al. 2020), has repeatedly purged deleterious mutations from the P. This would be similar to how rare recombination events between the sex chromosomes in European tree frogs have purged deleterious mutation from the Y chromosome (Guerrero et al. 2012). Future analyses will be able to quantify exact rates of recombination and gene conversion between haplotypes. However, the current data reveal indirect evidence of between-haplotype recombination, as only a fraction of SNPs within the supergene region were trans-species haplotype-specific based on the 19 M/P workers in our dataset (14.5%; 21,643/149,730 SNPs). The weak signature of genetic load on the P suggests that the lack of P/P individuals in *Formica* wood ants may be the result of a few mutations with highly deleterious effects rather than a general accumulation of deleterious mutations across the supergene.

## Conclusion

Supergenes are powerful study systems for revealing candidate genes controlling complex phenotypic traits. However, their non-recombining nature results in large haplotypes (often containing hundreds of genes) typically being inherited as a single unit, which makes it hard to pinpoint the causal genes. The retention of only two P-specific genes in the obligately polygynous species *F. aquilonia* and *F. paralugubris* has, conveniently, narrowed down the list of candidate genes significantly. Future studies will reveal the precise function of the *Zasp52* and *TTLL2* genes in *Formica* ants and their potential role in upholding the polygyny syndrome in obligately polygynous species lacking the full P haplotype.

## Materials and methods

### Supergene haplotype distributions of seven wood ant species

We analyzed Illumina paired-end data from 337 individuals within the wood ant complex. Of these samples, three are previously published haploid males with known supergene status (*n* = 1 *F. pratensis* with a P haplotype, *n* = 1 *F. truncorum* with a M haplotype, and *n* = 1 *F. truncorum* with a P haplotype; Table S1; Brelsford et al. 2020). The remaining samples are diploid workers (females) with unknown supergene distributions, of the following species: *F. aquilonia* (*n* = 101), *F. lugubris* (*n* = 23), *F. paralugubris* (*n* = 162), *F. polyctena* (*n* = 6), *F. pratensis* (*n* = 27), and *F. rufa* (*n* = 16) (Table S1). Of the 334 workers which are all collected from separate nests, 72 have been previously described and published (Portinha et al. 2022; Satokangas et al. 2023) while the remaining 262 samples are new for this study (Table S1). Details about DNA extraction method and sequencing technology for all non-published samples are in Table S1.

The data from the three males were trimmed using trimmomatic v0.39 (Bolger et al. 2014) with options “SLIDINGWINDOW:4:20 MINLEN:25 ILLUMINACLIP:adapters/TruSeq3-PE-2.fa:2:40:15”. All samples were then aligned to the *F. aquilonia*  *×*  *polyctena* reference genome (constructed from a haploid male hybrid individual; Nouhaud et al. 2022) using bwa mem v0.7.17, sorted using samtools sort v1.16.1 (Danecek et al. 2021) and deduplicated using picard MarkDuplicates v2.27 (http://broadinstitute.github.io/picard/). The resulting BAM files were filtered to only include reads mapping to chromosome 3 (Scaffold03) using samtools. We used deepvariant v1.4.0 (Poplin et al. 2018) to call variants for all BAM files separately (*n* = 145) with option –model_type WGS, and then performed joint variant calling using GLnexus v1.4.1-0-g68e25e5 (Yun et al. 2021) using options –config DeepVariantWGS and –trim-uncalled-alleles. We normalized the resulting VCF file using bcftools norm v1.16 and filtered variants using vcftools v0.1.17 (Danecek et al. 2011) with options –min-alleles 2 –max-alleles 2 –minQ 20 –minDP 3 –non-ref-ac 5 –max-missing 0.6 –mac 5 –remove-filtered-all. SNPs which were heterozygous in >90% of the samples were also removed using bcftools. We excluded samples with missing genotypes on ≥10% of called sites (*n* = 73), leaving 264 individuals (*n* = 261 workers; *n* = 3 males) with read depths between 9.1× and 69.0× (Table S2).

We calculated inbreeding coefficients (F_IS_ scores) for all samples using vcftools with option –het to identify samples that are supergene heterozygous (M/P). Of the 261 workers, 19 had extremely low F values (<–0.5), meaning that the observed homozygous sites were fewer than expected. These 19 samples (*n* = 5 *F. lugubris*, *n* = 3 *F. polyctena*, *n* = 8 *F. pratensis*, *n* = 3 *F. rufa*) were labeled as M/P genotypes (Table S1). To determine the supergene haplotype of the other samples, we then filtered the VCF file keeping sites where all 19 M/P workers were heterozygous while allowing <20% missing genotypes. This left 21,643 trans-species haplotype-specific SNPs. For all samples, we then counted the proportion of reference homozygous sites (0/0), heterozygous sites (0/1), and alternative homozygous sites (1/1) using bcftools v1.16 (Danecek et al. 2021) query with the flag -f “[%SAMPLE\t%GT\n]”.

### Species phylogeny

To construct a species phylogeny, we called joint variants from the deepvariant VCF files again using GLnexus (see above) for the 264 samples, this time with two additional outgroup individuals from *Formica exsecta* (Table S1). These outgroup samples were trimmed and aligned in the same way as the male samples. From the joint VCF file, we extracted SNPs from chromosome 3 outside of the supergene region (>10 kb away from chromosome ends and >200 kb away from the supergene region) using vcftools. We also excluded any genetic variants with heterozygosity in all 16 M/P individuals, leaving 40,851 SNPs. Bcftools was used to retain any biallelic SNPs, and the remaining 34,098 SNPs were filtered for minimum 50 bp distance using vcftools option –thin 50, leaving 12,776 SNPs. We used the script convert_vcf_to_nexus.rb (https://github.com/mmatschiner/tutorials/blob/master/species_tree_inference_with_snp_data/src/convert_vcf_to_nexus.rb; accessed 26th of June, 2024) to convert the VCF file to NEXUS format, from which we constructed a phylogenetic tree for all samples using the SVDquartets (Chifman and Kubatko 2014) algorithm, implemented in PAUP v4.0a168 (Swofford and Sullivan 2009). Default settings were used, except for raising the number of randomly analyzed quartets to 10 million (representing 4.9% of the total amount of quartets). We excluded 21 samples which did not cluster monophyletically in the resulting tree (Figure S7; *n* = 15 *F. aquilonia*, *n* = 6 F. lugubris) from the VCF file containing the 12,776 SNPs (see above) and re-converted the output VCF to NEXUS format. We then used SVDquartets to construct a species-level phylogeny using a taxon partition file, 1 million randomly sampled quartets (6.17% of the total number), and with the default number of bootstraps (*n* = 100).

### P-specific SNPs in obligately polygynous species with M/M genotypes

For each sample (*n* = 139), we counted the number of bi-allelic trans-species haplotype-specific SNPs (see above) occurring in CDS regions (*n* = 1,546 SNPS) with bcftools query using the flag -f “[%SAMPLE\t%GT\n]”. We interpreted a site as “P-specific” if the SNP genotype was either “0/1” or “1/1”. We used linear models in R v4.2.1 (R Core Team 2022) to test if any genes had significantly different numbers of P-specific SNPs between M/M workers of obligately polygynous species (*n* = 3) and socially polymorphic species (*n* = 3). We also calculated sequencing depth for each CDS region and all samples using bamstats04 from Jvarkit v36b5fa3 (Lindenbaum 2015). These values were normalized between samples, and the per-sample average was calculated for each gene. Two genes (“jg3505” and jg3507” in the *F. aquilonia*  *×*  *polyctena* gene annotation; Table S6) had significantly more P-specific SNPs in the obligately polygynous species.

We functionally annotated these two genes in the following way: First, we identified orthologs between the *F. aquilonia*  *×*  *polyctena* reference genome, two outgroup ant species (*Camponotus floridanus*, GCF_003227725.1; *Solenopsis invicta*, GCF_016802725.1), and a fruit fly (*Drosophila melanogaster*, GCF_000001215.4) using orthofinder v2.5.4 (Emms and Kelly 2019). Secondly, using the two resulting orthogroups (containing jg3505 and jg3507, respectively), we confirmed that all transcripts were orthologous also in the orthoDB v11 (Kuznetsov et al. 2023) database. And thirdly, we confirmed the gene structure by blasting the gene sequences, as well as those of adjacently positioned gene annotations, on the NCBI website. We found that jg3504 and jg3505 belong to the same gene (*Zasp52*; Table S6) and manually annotated these into a new gene annotation (*Zasp52*; Table S7), using additional evidence from downloaded amino acid sequences from orthoDB (accessed 12th of March; see orthogroup ID in Table S6).

The transcript jg3507, which belongs to the *TTLL2* gene (Table S6), and *Zasp52* are both putative P-specific duplications (see Results and Discussion). To see if the duplication covers the entire transcripts, we extracted sequencing depth values using jvarkit (as above) for all SNPs remaining after the quality filtering (see above) within CDS regions of these genes. We found that individuals carrying a P haplotype, and workers of obligately polygynous species with M/M genotypes, had higher sequencing depth across the entire *TTLL2* gene (Figures S3 and S4) but only across parts of the *Zasp52* gene (exons 10 to 16 in the curated transcript “Zasp52”; Figures S3 and S4; Table S7). We therefore created a separate transcript which contained only exons 10 to 16 and adjusted the first exon to include an additional eight codons where the first one was a start codon (“Zasp52-dup”; Figure S8; Table S7). These eight codons were included to aid the homology-based annotation (see below) and were not included in any of the results in the Results and Discussion section. Note also that the curated transcripts Zasp52 and Zasp52-dup contain the same trans-species haplotype-specific SNPs as the original transcript jg3505 (*n* = 22; Figure S8).

### Confirming duplication of Zasp52 and TTLL2 using de novo assemblies of *F. aquilonia* and *F. polyctena*

To verify that the P-specific copies of *Zasp52* and *TTLL2* are duplicated on the M haplotype in the obligately polygynous species, we aligned raw PacBio data from (i) the *F. aquilonia*  *×*  *polyctena* male from which the reference genome was constructed, (ii) five pooled *F. aquilonia* males, and (iii) five pooled *F. polyctena* males (Table S8), to the *F. aquilonia*  *×*  *polyctena* reference genome using pbmm2 v1.13.0 (https://github.com/PacificBiosciences/pbmm2) with option –preset SUBREAD. The aligned reads were sorted and indexed using samtools, and genetic variants were called across Scaffold03 using longshot v.0.4.1 (Edge and Bansal 2019). Of the 21,790 trans-species haplotype-specific SNPs (see above), only a small fraction of the SNPs from the PacBio data was heterozygotic or alternative homozygotic across the three datasets: 499 (2.3%) for the *F. aquilonia*  *×*  *polyctena* dataset, 687 (3.2%) for the *F. aquilonia* dataset, and 958 (4.4%) for the *F. polyctena* dataset. We therefore concluded that the *F. aquilonia* and *F. polyctena* data exclusively contain the M haplotype, similarly to the *F. aquilonia*  *×*  *polyctena* reference genome. We assembled the PacBio reads from all three datasets using Flye v.2.9.3 (Kolmogorov et al. 2019) with default parameters (genome statistics in Table S9). We used GeMoMa v1.6.4 (Keilwagen et al. 2019) to do homology-based annotations for all three de novo assemblies based on the annotation file for the *F. aquilonia*  *×*  *polyctena* reference genome (https://doi.org/10.6084/m9.figshare.14186522.v1). For the *F. aquilonia* assembly, the genes *Zasp52* and *TTLL2* each occurred as three gene copies on separate contigs (Table S10). We ran the pipeline purge_dups v1.2.5 (Guan et al. 2020) on this assembly and found that contig_1719 was a haplotig of contig_189, and gene annotations from this contig were not used for further analysis (Table S10).

From the amino acid sequences of *Zasp52* and *TTLL2* downloaded from OrthoDB (see above), we selected all ant species (*n* = 22) and removed short sequences (≥600 for *Zasp52* and ≥400 for *TTLL2*; cutoffs based on manual inspection) using seqkit v0.16.0 (Shen et al. 2016). For each gene, we aligned these 22 sequences together with the predicted proteins from GeMoMa using clustalo v1.2.4 (Sievers and Higgins 2018) with option -t Protein. The aligned sequences were manually inspected and then trimmed twice using trimAL v1.4.rev15 (Capella-Gutiérrez et al. 2009), first using the option -strictplus and then using the option -gt 0.8. Note that this trimming removed the first eight amino acids in the beginning of the first exon of the transcript Zasp52_dup, which were adjusted to include a start codon. The final alignments were 1080 (*Zasp52*) and 470 (*TTLL2*) amino acids long. We used iqtree2 v2.2.2.7 (Minh et al. 2020) to create phylogenetic trees from the trimmed alignments using options -m LG+F+G -nt AUTO. We calculated pairwise sequence identity between all sequences using a custom python script. We determined which gene copies in the de novo assemblies were the duplicated paralogs based on the topology between the sequences and sequence similarity to the *F. aquilonia*  *×*  *polyctena* reference genome (Fig. 4; Table S10).

We trimmed, aligned, and called variants from *n* = 5 published *Formica* samples outside the wood ant complex (Table S1) using the same method as for the other Illumina samples (see above). We extracted SNPs at the positions of the wood ant trans-species haplotype-specific SNPs occurring in CDS regions using bcftools.

### Mutation load

The genome assembly GFF3 file was transformed to GTF2.2 format using the script agat_convert_sp_gff2gtf.pl v.1.0.0 from agat v1.0.0 (Dainat), and protein and CDS sequence files were created from this file using gffread v0.12.7 (Pertea and Pertea 2020). A snpEff v5.2 (Cingolani et al. 2012) database was build based on these files. From the filtered VCF file containing 139 individuals (see above), we extracted SNPs from each worker using vcftools and the option –keep <IND>–mac 1. The functional effects of all SNPs were predicted using snpEff ann. From this output, we retained genetic variants from primary transcripts with high, moderate, and low predicted effects.

### Alignment, variant calling, and expression levels of RNA-seq data

We indexed the *F. aquilonia*  *×*  *polyctena* and the *F. aquilonia* reference genomes using STAR v2.7.11a (Dobin et al. 2013) with option –sjdbOverhang 149. We aligned the RNA data from a pool of 400 *F. aquilonia* workers (read length 150 bp; this study) to both reference genomes using STAR with option –twopassMode Basic. The reads were sorted and indexed using sambamba v0.7.0 (Tarasov et al. 2015), duplicates were marked using picard MarkDuplicates v2.27.5, and GATK SplitNCigarReads v4.5.0.0 (Van der Auwera et al. 2000) was used to split reads. We called variants for each sample separately using GATK Haplotypecaller. We counted aligned reads per gene using featureCounts v.2.0.1 (Liao et al. 2014) and calculated transcripts per million (TPM) per gene using the following formula:


((genereadcount)/(genelength))/((totalreadcount)/(1e^6))

We defined the expression level of genes based on the cutoff values used by the EMBL-EBI Expression Atlas (last accessed 20 March 2024).

## Supplementary Material

msaf320_Supplementary_Data

## Data Availability

Supporting BAM files for all wood ant samples are uploaded to Zenodo (10.5281/zenodo.17466022). Other supporting sequences are available in the NCBI Sequence Read Archive (SRA) under BioProject accession no. PRJNA1358004. Supplementary code is available on GitHub at https://github.com/hsigeman/wood-ant-supergene_MBE_2026.
