# Supplementary material for: The loss of a supergene in obligately polygynous Formica wood ant species
Source: Mol Biol Evol. 2025 Dec 16;42(12):msaf320. doi: 10.1093/molbev/msaf320 (PMC12728502; doi:10.1093/molbev/msaf320)
Supplement: msaf320_Supplementary_Data [file msaf320_supplementary_data.zip › Sigeman et al. 2025 Supplementary figures.pdf]

Supplementary Figures S1-S8 and Supplementary Note

The loss of a supergene in obligately polygynous *Formica* wood ant species

Hanna Sigeman[1,2], Ina Satokangas[3], Matthieu De Lamarre[4], Patrick Krapf[3], Pierre Nouhaud[3,5], Riddhi Deshmukh[4], Heikki Helanterä[1], Michel Chapuisat[4], Jonna Kulmuni[3], and Lumi Viljakainen[1]

[1]Ecology and Genetics Research Unit, University of Oulu, Finland
[2]Department of Medical Biochemistry and Microbiology, Uppsala University, Sweden
[3]Organismal and Evolutionary Biology Research Programme, University of Helsinki, Finland
[4]University of Lausanne, Switzerland
[5]University of Montpellier, France

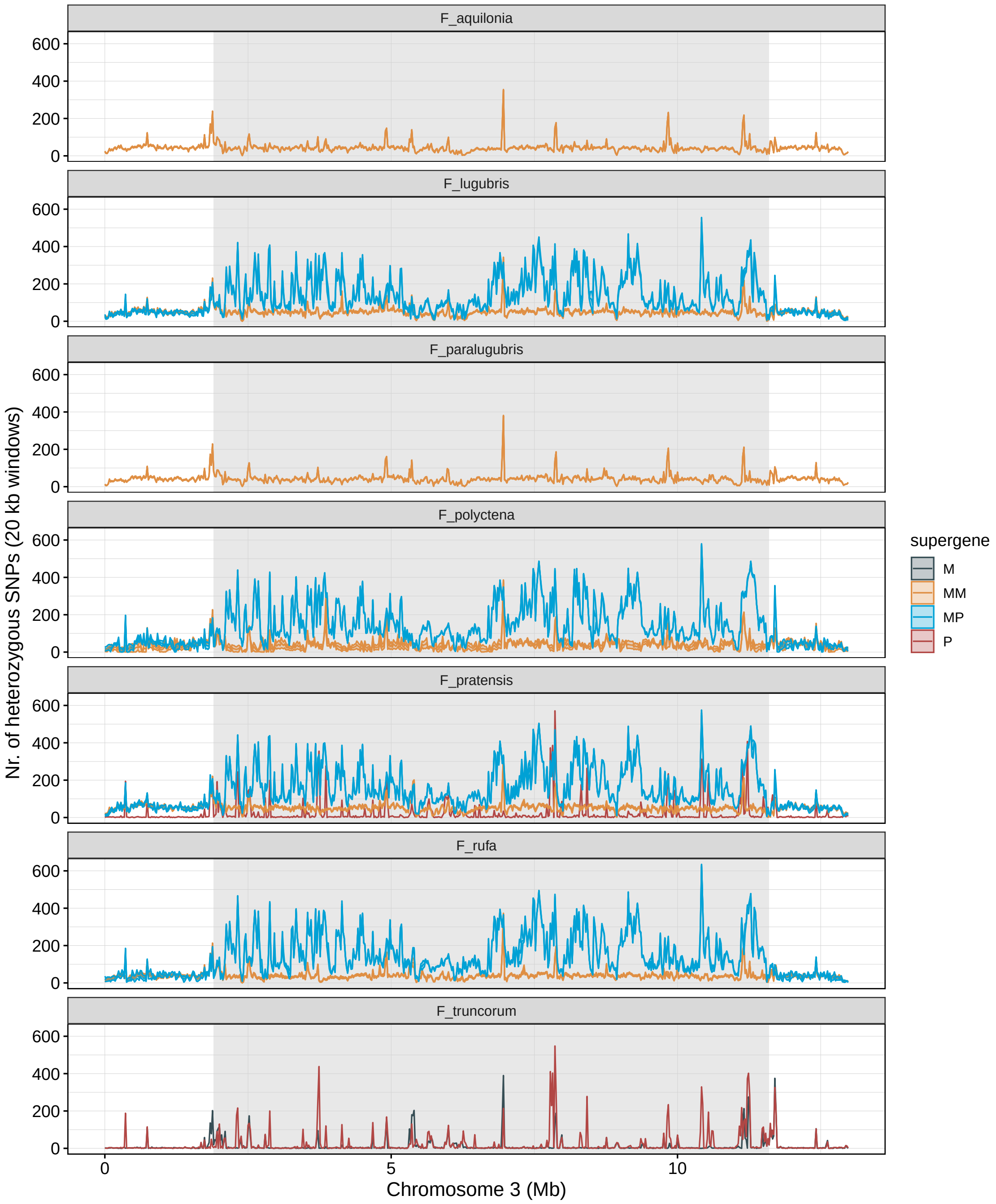

Figure S1: Mean values (±SE) of heterozygous SNPs (0/1) across 20 kb windows, plotted separately per species and supergene haplotype.

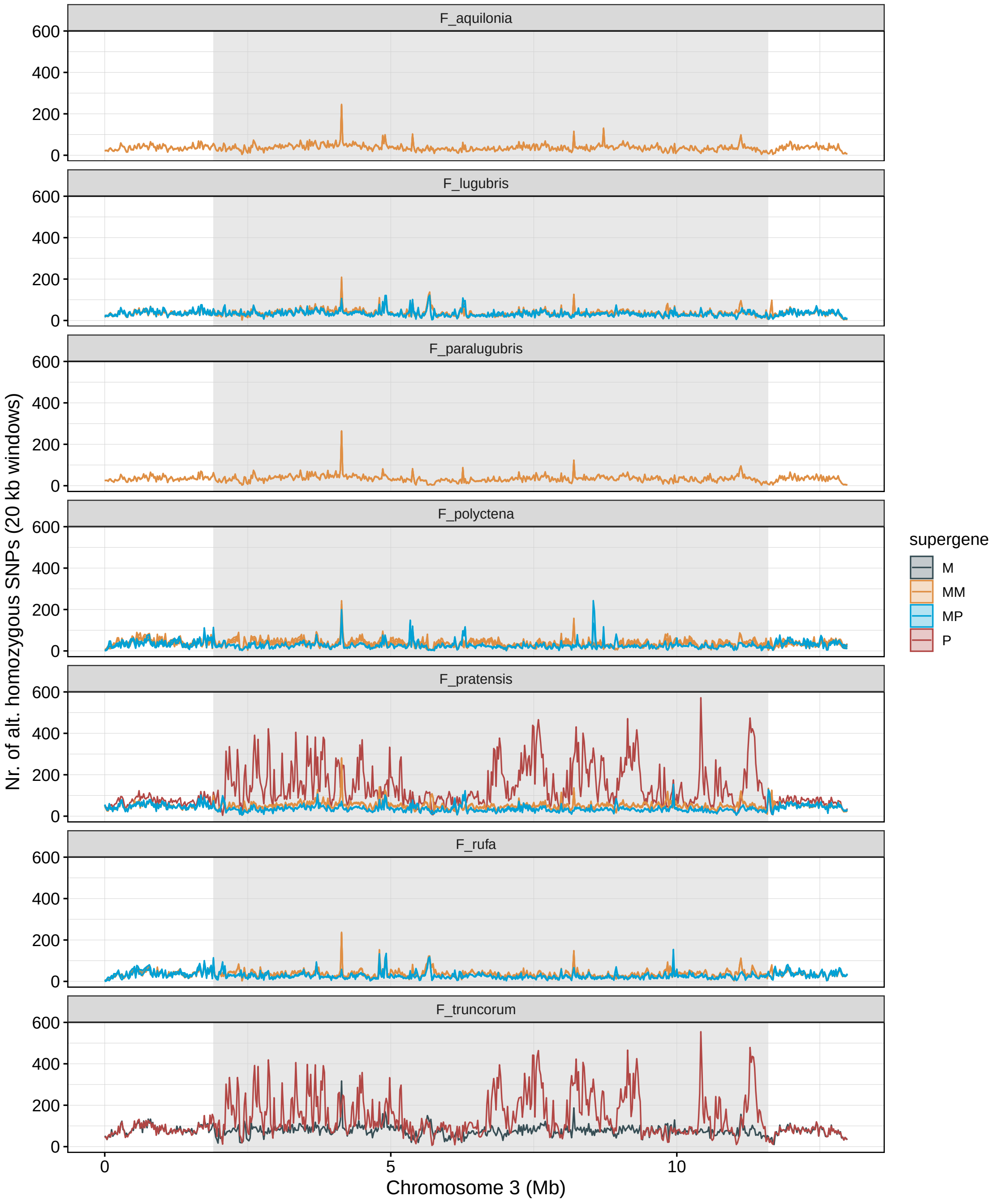

Figure S2: Mean values (±SE) of alternative homozygous SNPs (1/1) across 20 kb windows, plotted separately per species and supergene haplotype.

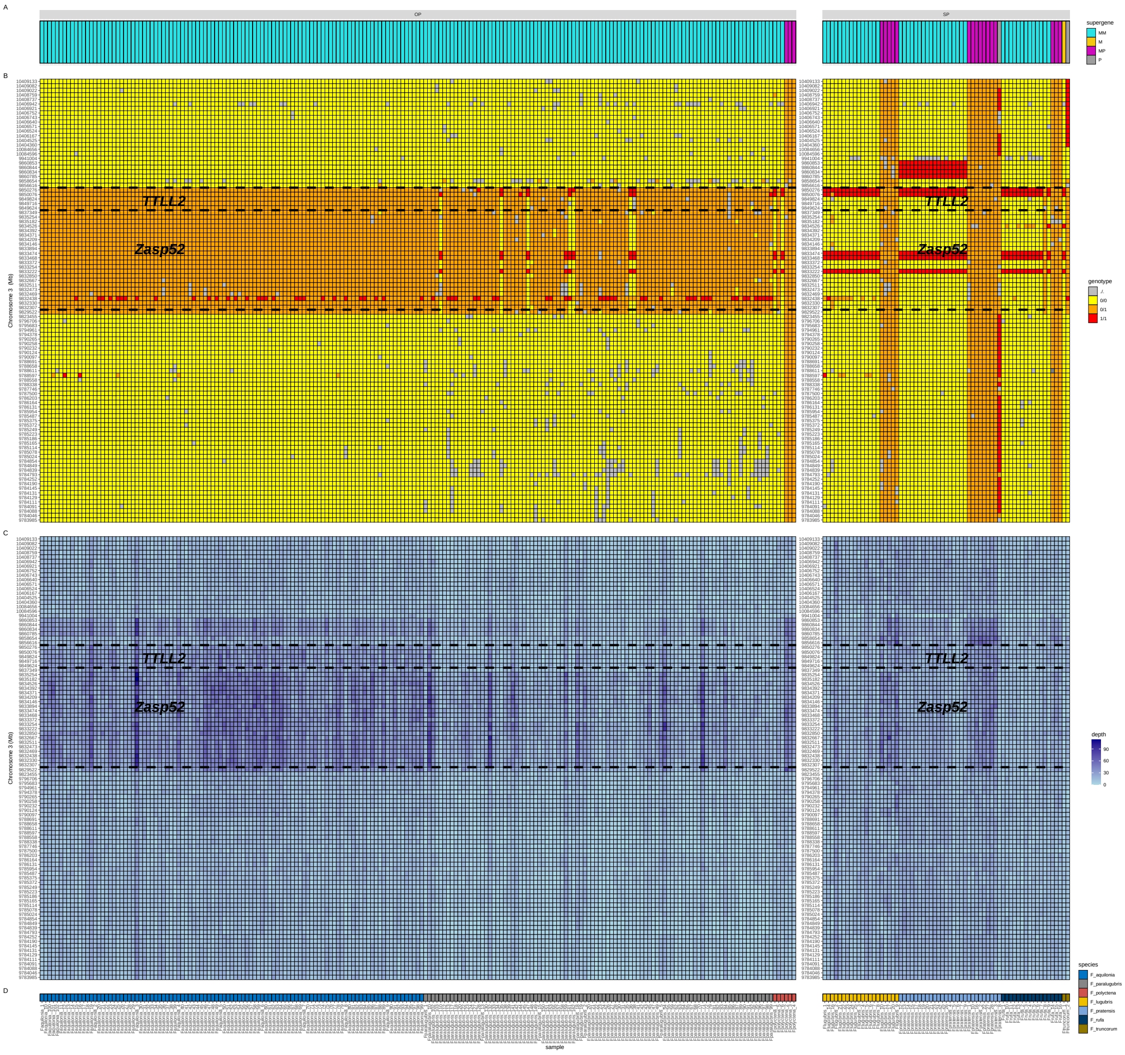

Figure S3: Heatmap showing (**A**) supergene genotype, (**B**) genotypes at the trans-species haplotype-specific SNPs in the genome region around *Zasp52* and *TTLL2* (Scaffold03:9782000-10410000, (**C**) sequencing depth values for the same genome region, and (**D**) species. OP: obligate polygynous, SP: socially polymorphic

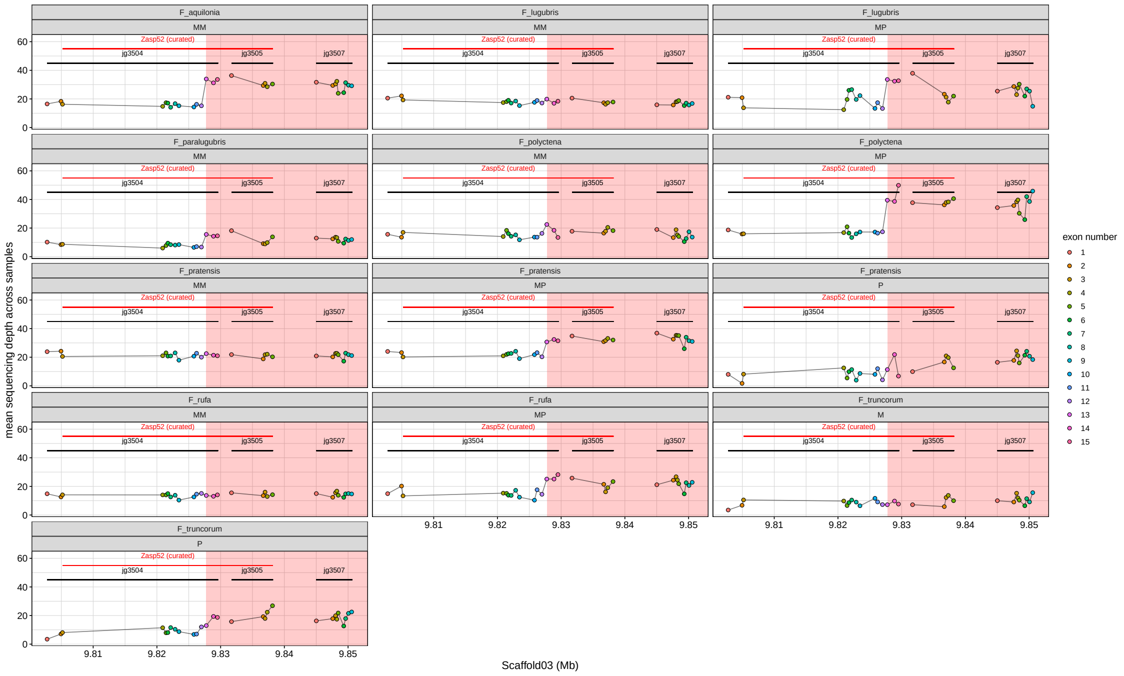

Figure S4: Per-CDS sequencing depth values for the genes *Zasp52* (jg3504 and jg3505) and *TTLL2* (jg3507). The values are averages across all basepairs in the region. The black lines mark the extent of the original transcripts and the red line show the extent of the manually curated *Zasp52* gene annotation. The red background marks the region hypothesized to be duplicated in individuals with high heterozygosity at these genes (see Figure S3).

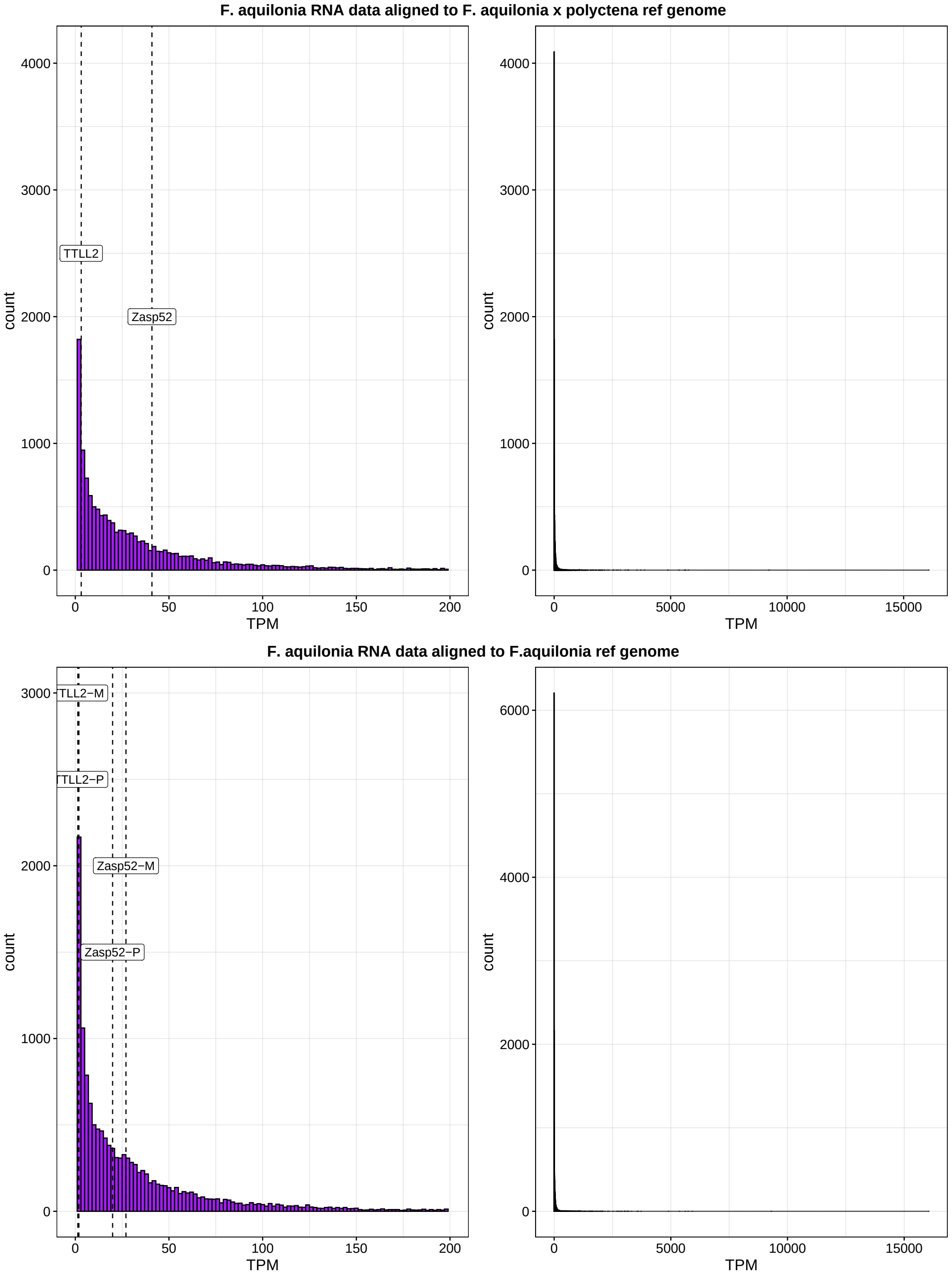

Figure S5: TPM values for the genes TTLL2 and Zasp52, based on RNAseq data from 400 F. aquilonia workers aligned to (top) the *F. aquilonia x polyctena* and (bottom) the de novo *F. aquilonia* reference genome. The left-side panels show TPM values ≤ 200 (for increased visibility) while the right-side panels show all values.

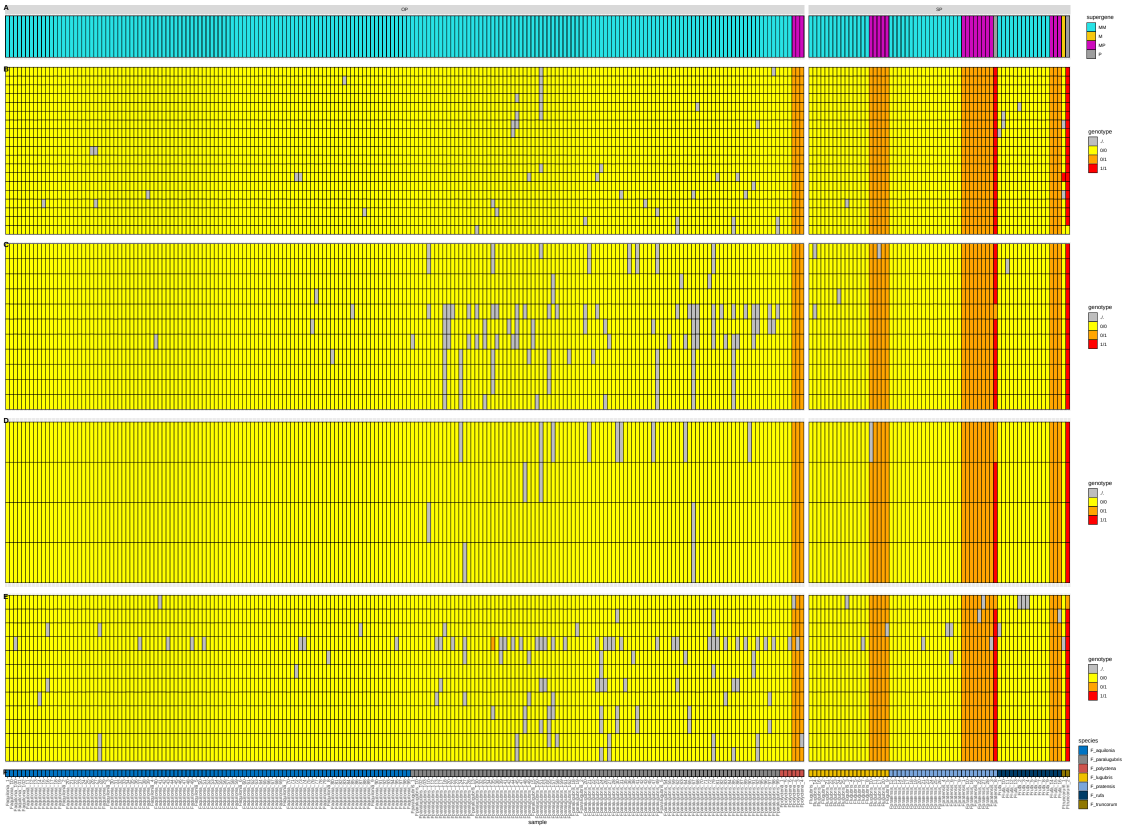

Figure S6: Heatmap showing (**A**) supergene genotype, and genotypes at the trans-species haplotype-specific SNPs at the genes (**B**) *Knockout*, (**C**) *Single-minded*, (**D**) *ZPF148*, and (**E**) *AmGR10*. The last row shows (**F**) Sample ID's and species. OP: obligate polygynous, SP: socially polymorphic

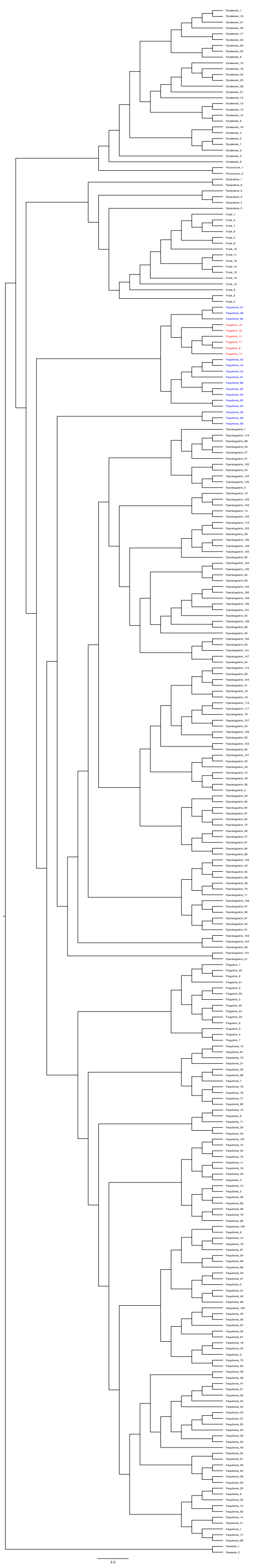

Figure S7: Output tree from SVDquartets of all 264 wood ant samples and two outgroup *F. excecta* samples (Table S1). The highlighted samples (i.e., non-black tip labels) were removed from the input VCF file before constructing the final species tree (Figure 2a; see Methods).

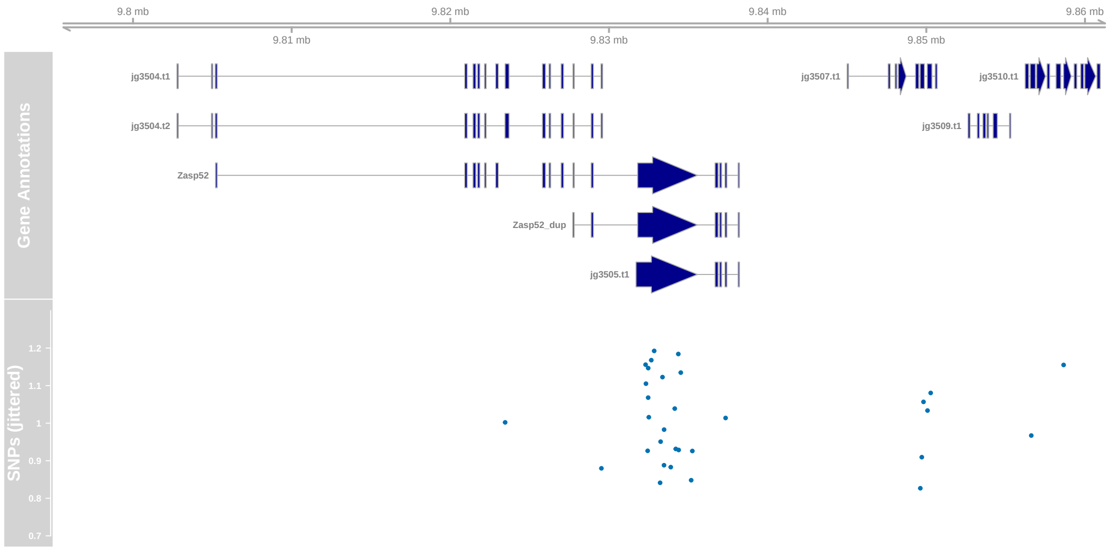

Figure S8: Original and curated gene models of *Zasp52* and *TTLL2* (see Main Text for details). The gene models starting with "jg" are from the *Formica aquilonia x polyctena* gene annotation, while the others are manually curated (provided in Table S7). P-specific SNP's are shown on the bottom panel, jittered along the y-axis for increased visibility.

Supplementary Note

A parallel study to ours with partially overlapping results was recently published by Lagunas-Robles et al. in *JEB* [1]. In this study, the authors also find that *F. paralugubris* lacks the P haplotype, but that workers in one out of four studied *F. aquilonia* nests carry it. The authors categorized the three *F. aquilonia* nests with only M/M workers as monogynous based on between-worker relatedness, while the fourth was classified as polygynous. Since these results are contradictory to our study—where we describe *F. aquilonia* as obligately polygynous and lacking the P haplotype—we provide this Supplementary Note to discuss these differences.

Many studies have, based on within-colony relatedness and observations of multiple queens, described *F. aquilonia* as obligately polygynous [2, 3, 4, 5, 6, 7, 8]. For example, a review of genetic studies on *Formica* species by Sundström et al. [7] describes relatedness in *F. aquilonia*, *F. paralugubris*, and *F. polyctena* as "invariably low indicating the obligate presence of many reproductively active queens. Indeed, excavations and calculations based on genetic data indicate that the queen number frequently rises to several hundreds". Of the 14 *Formica* species included in the review, *F. aquilonia* had the lowest within-colony relatedness (mean $r = 0.02 \pm 0.08$) [7]. It is therefore a highly unexpected finding that three out of four studied *F. aquilonia* nests were classified as monogynous in Lagunas-Robles et al. [1].

Based on the discrepancies in social colony organization between the *F. aquilonia* samples in Lagunas-Robles et al. [1] and other studies [2, 3, 4, 5, 6, 7, 8], we hypothesized that the *F. aquilonia* samples in Lagunas-Robles et al. [1] were either sampled from a hybrid population or that the species was misclassified.

To evaluate the species identification of *F. aquilonia* in Lagunas-Robles et al. [1], we downloaded a VCF file provided as Supplementary Data which included all RADseq data from this study as well as whole-genome sequencing data from two *F. truncorum* males:
`RADs_wTrunc_wg_dp8_maxmiss75_2alleles_mac2_noindels.recode.vcf`

We also downloaded previously published whole-genome sequencing data from species in the *Formica rufa* complex. These included 2 *F. truncorum* males (same as used in Lagunas-Robles et al. [1]), 10 *F. polyctena* workers, 10 *F. aquilonia* workers, 2 *F. rufa* workers, and 1 *F. lugubris* worker (Table S12). We aligned these to the *Formica aquilonia x polyctena* reference genome, along with unpublished whole-genome data from *F. aquilonia* ($n = 20$), *F. paralugubris* ($n = 5$), and *F. lugubris* ($n = 10$) (Table S12). These samples were aligned using the same pipeline as described in the Methods section. For increased comparability to the RADseq data from Lagunas-Robles [1], we used the `bcftools` v1.16 [10] algorithms `bcftools mpileup` (with options `--min-MQ 20 -a FORMAT/DP`) and `bcftools call` to score variants at the same sites as in the RADseq VCF file (n = 31,063 sites).

After variant calling, we filtered the VCF using `vcftools` [9] for biallelic sites with a minimum depth of 8x and removed indels and any sites on Scaffold03 (`--minDP 8 --remove-indels --not-chr Scaffold03 --min-alleles 2 --max-alleles 2`). We then merged the two VCF files by identifying shared positions and genotype calls with `bcftools isec`, extracting those positions with `bcftools view`, and merging them using `bcftools merge`. This process retained 17,786 of the original 31,063 sites. We calculated the proportion of missing sites per individual using `vcftools` and removed those that had missing genotypes at more than 30% of the sites ($n = 22$). Lastly, we removed SNPs with more than 10% missing data among the remaining individuals, resulting in 11,475 SNPs.

We assigned the remaining samples to the following groups (see Table S12):

a. Formica_aquilonia: Whole-genome sequencing data from published and unpublished *F. aquilonia* samples.

b. Formica_polyctena: Whole-genome sequencing data from published *F. polyctena* samples.

c. Formica_truncorum: Whole-genome sequencing data from a published *F. truncorum* male (the other one was filtered out due to a high proportion of missing genotypes).

d. Formica_lugubris: Whole-genome sequencing data from published and unpublished *F. lugubris* samples.

e. Formica_paralugubris: Whole-genome sequencing data from published and unpublished *F. paralugubris* samples.

f. Formica_paralugubris_RADSEQ: RADseq data from Lagunas-Robles [1].

g. Formica_aquilonia_RADSEQ: RADseq data from Lagunas-Robles [1].

h. Formica_truncorum_RADSEQ: RADseq data from Lagunas-Robles [1].

i. Formica_truncorum2: Whole-genome sequencing data from one same *F. truncorum* male as above (Formica_truncorum), included in the VCF file from Lagunas-Robles[1].

We evaluated the species identification in four ways:

1. Phylogenetic tree using SVDquartets

We constructed a phylogenetic tree with `SVDquartets` v4.0a [11] (see Methods), based on 100,000 quartets and with the *F. truncorum* samples as outgroups. The RADseq *F. aquilonia* samples (Formica_aquilonia_RADSEQ) formed a sister-group to the whole-genome sequenced *F. aquilonia* samples (Formica_aquilonia).

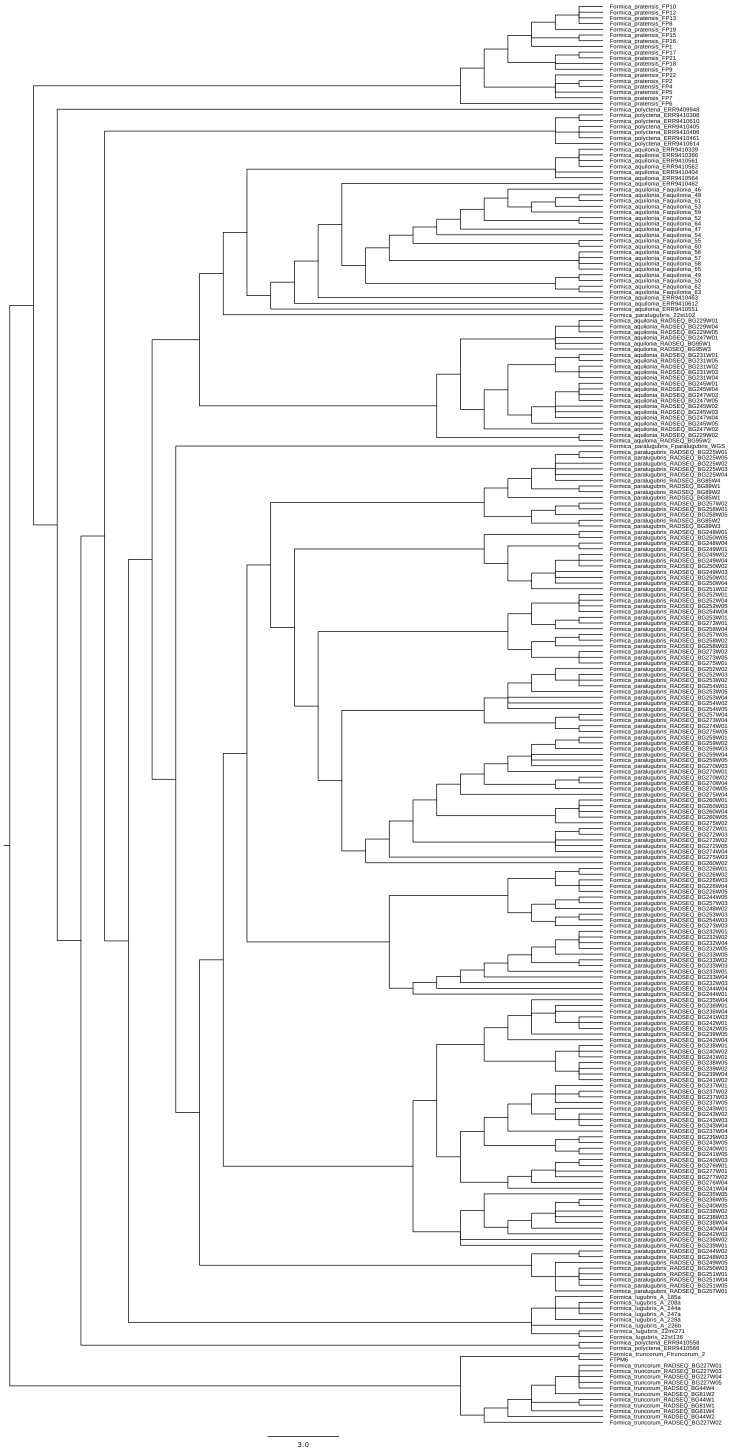

Supplementary Note Figure 1: Phylogenetic tree with bootstrap support values. Tip labels show concatenated group names and sample IDs. See Table S12 for detailed sample information.

2. PCA

Using `plink2` v2.0.0 [12], we transformed the VCF file to BED format and ran a PCA analysis with 10 principal components. The results were visualized by plotting PC1 vs. PC2. The RADseq *F. aquilonia* (Formica_aquilonia_RADseq) samples and the whole-genome sequencing *F. aquilonia* samples (Formica_aquilonia) formed separate clusters.

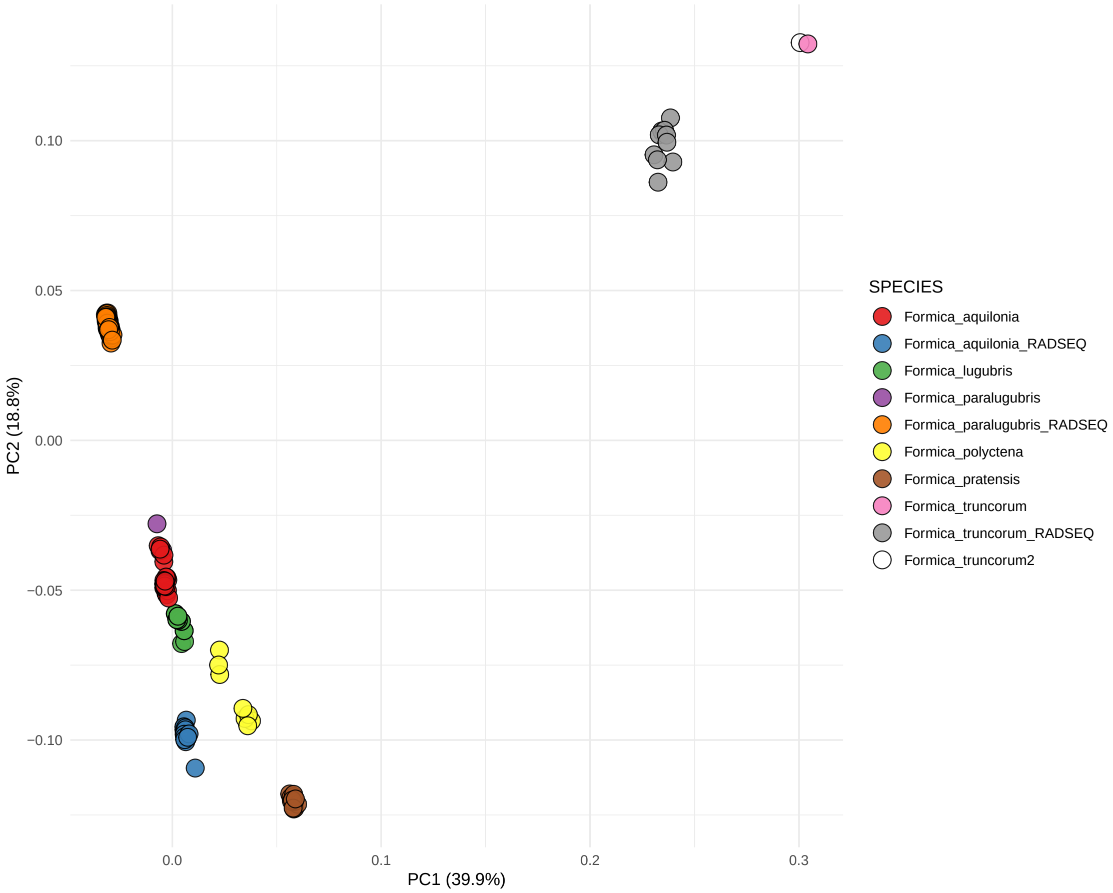

Supplementary Note Figure 2: PCA analysis. See Table S12 for sample information for all groups.

3. Discriminant Analysis of Principal Components

We did a Discriminant Analysis of Principal Component (DAPC) analysis using the `adegenet` package [13] in `R`, without using prior species information. We retained 200 PCs and chose to divide the data into 5 clusters based on the BIC scores. All 6 eigenvalues were retained. The RADseq *F. aquilonia* (Formica_aquilonia_RADseq) samples and the whole-genome sequencing *F. aquilonia* samples (Formica_aquilonia) grouped into separate clusters.

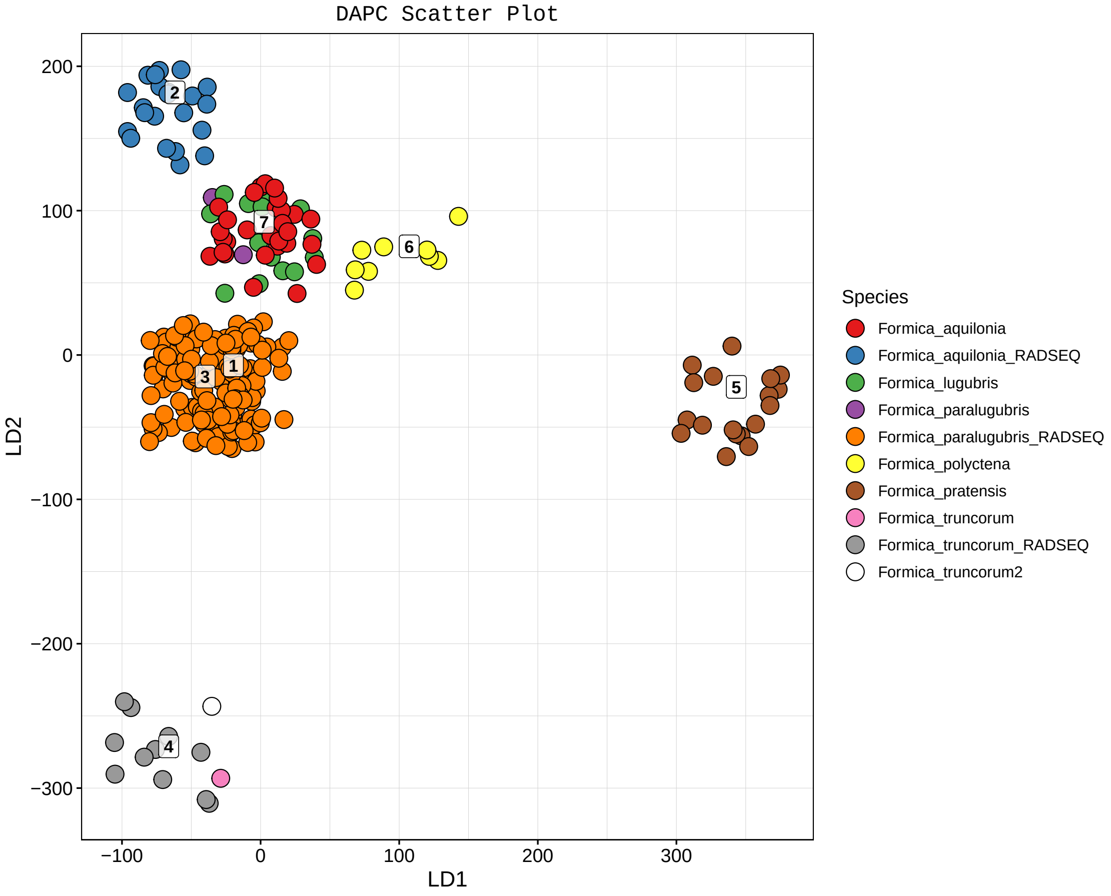

Supplementary Note Figure 3: DAPC analysis. The datapoints within each cluster are jittered along both the x-axis and y-axis for increased visibility. See Table S12 for sample information for all groups.

4. Estimation of individual ancestry coefficients

We estimated individual ancestry coefficients using the `snmf` algorithm from the `LEA` package [14] in `R`. The RADseq *F. aquilonia* (Formica_aquilonia_RADseq) samples and the whole-genome sequencing *F. aquilonia* samples (Formica_aquilonia) grouped into separate clusters.

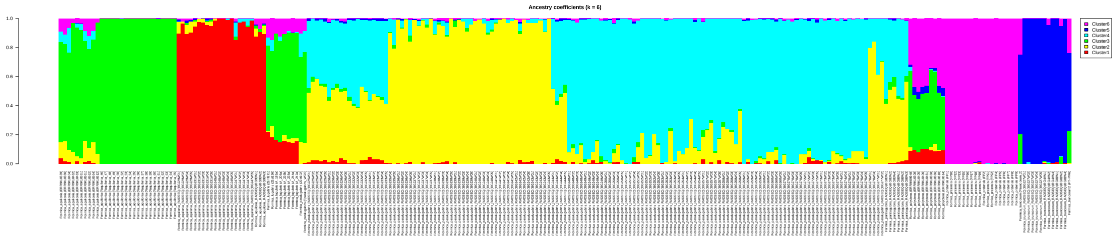

Supplementary Note Figure 4: Individual ancestry coefficient analysis with K = 6 clusters. The labels on the x-axis show concatenated group names and sample IDs. See Table S12 for detailed sample information.

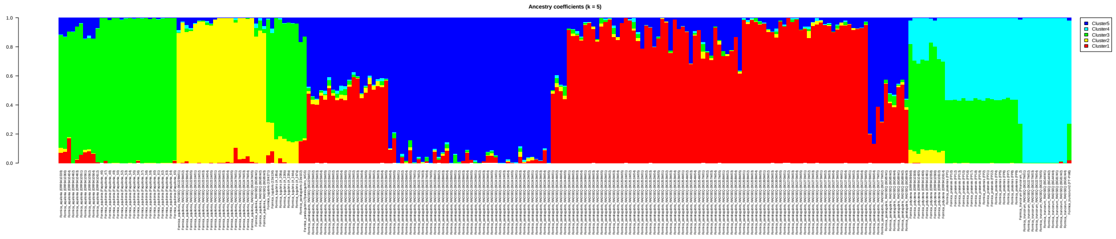

Supplementary Note Figure 5: Individual ancestry coefficient analysis with K = 5 clusters. The labels on the x-axis show concatenated group names and sample IDs. See Table S12 for detailed sample information.

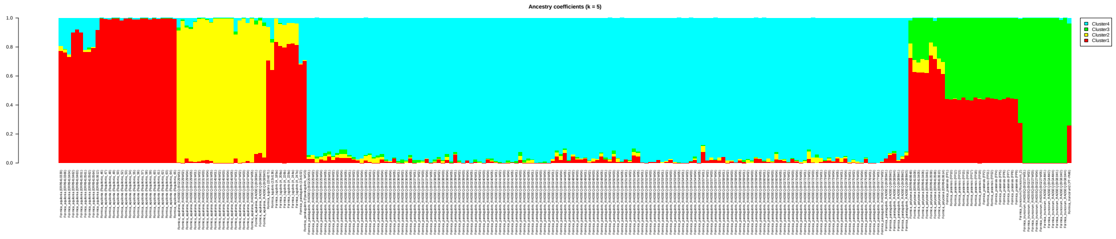

Supplementary Note Figure 6: Individual ancestry coefficient analysis with K = 4 clusters. The labels on the x-axis show concatenated group names and sample IDs. See Table S12 for detailed sample information.

Conclusion

In three of these four analyses (Supplementary Note Figures 2-6), the RADseq sequenced *F. aquilonia* samples clustered separately from the whole-genome sequenced *F. aquilonia* samples, while in the phylogenetic tree they formed sister-groups (Supplementary Note Figure 1). We cannot exclude the possibility that differences in sequencing technology, or the relatively low number of SNPs analysed here (n = 11,475), are influencing these results. However, we believe that a more likely explanation is that the RADseq *F. aquilonia* samples are truly genetically distinct from other sequenced *F. aquilonia* samples. Based on the mainly monogynous social

colony structure of the RADseq *F. aquilonia* samples [1] which is not characteristic of *F. aquilonia* [2, 3, 4, 5, 6, 7, 8], and the genetic distance between these and the whole-genome sequenced *F. aquilonia* samples (Supplementary Note Figures 1-6), we believe these samples may originate from a hybrid population, which are common in nature, or have been misclassified. Therefore, we do not consider the results of the Lagunas-Robles et al. study, in which the P haplotype was found among samples assigned to *F. aquilonia* [1], to be contradictory to our own findings. Further investigation would help clarify the taxonomic status of these disjunct *Formica* populations.

References

[1] Lagunas-Robles G, et al. (2025) Unexpected absence of a multiple-queen supergene haplotype from supercolonial populations of Formica ants. *J Evol Biol* 38(4):543-553.

[2] Pamilo P. (1982) Genetic population structure in polygynous Formica ants. *Heredity* 48:95-106.

[3] Rosengren R, Pamilo P. (1983) The evolution of polygyny and polydomy in mound-building Formica ants. *Acta Entomol Fenn* 42:65-77.

[4] Pamilo P, et al. (1992) Genetic differentiation of disjunct populations of the ants Formica aquilonia and Formica lugubris in Europe. *Insectes Sociaux* 39:15-29.

[5] Mäki-Petäys, H., et al. (2005) Genetic changes associated to declining populations of Formica ants in fragmented forest landscape. *Mol Ecol* 14:733-742.

[6] Pamilo P, et al. (2005) Genetic patchwork of network-building wood ant populations. *Annales Zoologici Fennici* 42:179-187.

[7] Sundström L, et al. (2005) Genetic population structure and dispersal patterns in Formica ants - a review. *Annales Zoologici Fennici* 42:163-177.

[8] Seifert B. (2018) The ants of Central and North Europe. *Lutra.*

[9] Danecek P, et al. (2011) The variant call format and VCFtools. *Bioinformatics* 27(15):2156-2158.

[10] Danecek P, et al. (2021) Twelve Years of SAMtools and BCFtools. *GigaScience* 10(2):1-4. Danecek m.fl., ?Twelve Years of SAMtools and BCFtools?.

[11] Chifman J, Kubatko L. (2014) Quartet Inference from SNP Data Under the Coalescent Model. *Bioinformatics* 30(24):3317-3324.

[12] Chang et al. (2015) Second-generation PLINK: rising to the challenge of larger and richer datasets. *GigaScience* 4:7

[13] Jombart T. (2008) adegenet: a R package for the multivariate analysis of genetic markers. *Bioinformatics* 24(11):1403-1405.

[14] Frichot E, François O. (2015) LEA: An R package for landscape and ecological association studies. *Methods in Ecology and Evolution* 6(8)-925-929.
